# The role of *IREB2 *and transforming growth factor beta-1 genetic variants in COPD: a replication case-control study

**DOI:** 10.1186/1471-2350-12-24

**Published:** 2011-02-14

**Authors:** Sally L Chappell, Leslie Daly, Juzer Lotya, Aiman Alsaegh, Tamar Guetta-Baranes, Josep Roca, Roberto Rabinovich, Kevin Morgan, Ann B Millar, Seamas C Donnelly, Vera Keatings, William MacNee, Jan Stolk, Pieter S Hiemstra, Massimo Miniati, Simonetta Monti, Clare M O'Connor, Noor Kalsheker

**Affiliations:** 1 The University of Nottingham. School of Molecular Medical Sciences, University Hospital, Queens Medical Centre, Nottingham, NG7 2UH, UK; 2 University College Dublin. UCD School of Public Health & Population Science, UCD, Belfield, Dublin 4, Ireland; 3 Hospital Clinico y Provincial de Barcelona. Service de Pneumologia, Hospital Clinic, Villarroel, 170, 08036, Barcelona, Spain; 4 University of Bristol. Lung Research Group, Department of Clinical Science at North Bristol, Southmead Hospital, Westbury on Trym, Bristol BS10 5NB, UK; 5 University College Dublin. UCD School of Medicine and Medical Science, The Conway Institute, UCD, Belfield, Dublin 4, Ireland; 6 Letterkenny General Hospital, Letterkenny, Co. Donegal, Ireland; 7 ELEGI Colt Laboratories, MRC Centre for Inflammation Research, Level 2, Room C2.29, The Queen's Medical Research Institute, 47 Little France Crescent, Edinburgh EH16 4TJ, UK; 8 Leiden University Medical Center, Department of Pulmonology (C3-P), Albinusdreef 2, P.O. Box 9600, 2300 RC Leiden, The Netherlands; 9 CNR Institute of Clinical Physiology, Via G. Moruzzi 1-56124, Pisa, Italy

## Abstract

**Background:**

Genetic factors are known to contribute to COPD susceptibility and these factors are not fully understood. Conflicting results have been reported for many genetic studies of candidate genes based on their role in the disease. Genome-wide association studies in combination with expression profiling have identified a number of new candidates including *IREB2*. A meta-analysis has implicated transforming growth factor beta-1 (*TGFbeta1*) as a contributor to disease susceptibility.

**Methods:**

We have examined previously reported associations in both genes in a collection of 1017 white COPD patients and 912 non-diseased smoking controls. Genotype information was obtained for seven SNPs in the *IREB2 *gene, and for four SNPs in the *TGFbeta1 *gene. Allele and genotype frequencies were compared between COPD cases and controls, and odds ratios were calculated. The analysis was adjusted for age, sex, smoking and centre, including interactions of age, sex and smoking with centre.

**Results:**

Our data replicate the association of *IREB2 *SNPs in association with COPD for SNP rs2568494, rs2656069 and rs12593229 with respective adjusted p-values of 0.0018, 0.0039 and 0.0053. No significant associations were identified for *TGFbeta1*.

**Conclusions:**

These studies have therefore confirmed that the *IREB2 *locus is a contributor to COPD susceptibility and suggests a new pathway in COPD pathogenesis invoking iron homeostasis.

## Background

Chronic obstructive pulmonary disease (COPD) is a leading cause of morbidity and mortality, and is predicted to become the 4^th ^leading cause of death by the year 2030 [1]. Whilst smoking is a significant environmental cause of COPD, not all smokers will develop disease. It is well recognised that COPD has a genetic component as well as environmental, and that this may account for these differences in susceptibility.

Many candidate gene studies have been carried out over the past few years, with varying degrees of reproducibility. Conflicting results may be due to population differences, spurious results caused by small sample sizes and the subsequent low power of the study to detect true associations or to variation in the phenotype. Meta-analysis can be used to pool results from genetic studies and give an overall conclusion, and this approach has been used to summarise the results for several COPD candidates [2]. This study revealed significant associations for three polymorphisms in transforming growth factor beta 1 (*TGFB1*), although it was acknowledged that this was a limited analysis based on a small number of studies.

Other strategies for identifying the genetic basis of complex human disease include integrative approaches which combine gene expression data with association studies, or unbiased genome-wide approaches. Both of these strategies have also been employed within the study of COPD.

A recent genome-wide association study highlighted a region on chromosome 15q25 which shows strong association to COPD [3]. This region contains several genes, including members of the nicotinic acetylcholine receptors (*CHRNA5*, *CHRNA3 *and *CHRNB4*) and the iron regulatory protein 2 (*IREB2*). Although this region has also been linked to lung cancer and nicotine addiction as well as COPD [4,5], the strong levels of linkage disequilibrium make it difficult to refine the location of the true association signal and the functional variants. However, the expression of *IREB2 *has also been shown to be altered in lung tissue from COPD patients compared to controls [6], adding to the evidence for *IREB2 *acting as a COPD susceptibility gene.

The aim of the current study was to investigate polymorphisms in the *TGFB1 *and *IREB2 *genes in our existing large collection of COPD cases and controls.

## Methods

### Subjects

COPD cases and control subjects were recruited at six European centres. Numbers from each centre were: Barcelona - 70 controls and 138 cases; Bristol - 152 controls and 129 cases; Dublin - 195 controls and 196 cases; Edinburgh - 81 controls and 168 cases; Leiden - 216 controls and 188 cases and Pisa - 198 controls and 198 cases. Approval for the study was obtained from the appropriate committees at each recruitment centre. Informed consent was obtained from all subjects. Criteria for patient recruitment were a firm clinical diagnosis of stable COPD; airflow limitation as indicated by FEV_1 _≤70% normal predicted values and FEV_1_/FVC <70%; no significant reversibility on bronchodilation and a smoking history of ≥20 pack years. Patients were excluded from the study if they had an established diagnosis of asthma, lung cancer, a history of atopy, known AAT deficiency or a serum AAT level of less than 1.0 g/L. They were also excluded if they had had an acute exacerbation in the 4 weeks preceding assessment for the study. Disease severity was classified according to GOLD as follows: GOLD Stage II (moderate disease): FEV1/FVC ratio of <0.70 and FEV1 predicted ≥50% and <80%; GOLD Stage III (severe disease): FEV1/FVC ratio of <0.70 and FEV1 predicted ≥30% and <50%; and GOLD Stage IV (very severe disease): FEV1/FVC ratio of <0.70 and FEV1 predicted <30% [7].

Control subjects were recruited at each centre to match COPD patients for age, gender and smoking history. Exclusion criteria were as described for cases and also included a family history of COPD. Only individuals with no evidence of airflow obstruction (FEV_1 _and FVC ≥80% and FEV_1_/FVC >70%) were included in the control group. Only white Caucasians were recruited for cases and controls. Complete matching between cases and controls was not achieved, but this was taken into account during the analysis.

### Genotyping of Study Samples

Four SNPs were chosen for *TGFB1*, which were the same as the ones included in the recent meta-analysis [2]. These were rs2241712, rs1800469, rs1800470 (formerly known as rs1982073) and rs6957. Seven SNPs were included for *IREB2*, as identified by a recent publication [6]. These were rs2568494, rs2656069, rs1964678, rs12593229, rs10851906, rs965604 and rs13180. Genotyping was carried out commercially by K-Bioscience (Hertfordshire, UK). As a quality control measure, approximately 5% of samples were genotyped in duplicate to check for concordance. In addition, a selection of samples were also genotyped using restriction enzyme digestion, allele-specific PCR or direct sequencing to confirm the genotyping results from K-Bioscience.

### Statistical Analysis of Genetic variation

Each of the SNPs in the IREB2 and TGF1 genes was analyzed for Hardy-Weinberg equilibrium (HWE) using SAS/Genetics PROC ALLELE software [8]. HWE analysis was performed on all controls and by each centre. The analysis of allele and genotype frequencies in cases and controls was performed using the same program. The p-values and odds ratios for genotype and allele frequencies are obtained by using SAS/Genetics PROC LOGISTIC software. Odds ratios for individual SNP allele distributions are relative to the common allele and for genotypes they are relative to the major homozygous genotype. The adjusted p-values and odds ratios with 95% confidence intervals for genotype and allele frequencies adjust for age, sex, smoking and centre, including interactions of age, sex and smoking with centre. This adjustment was done to eliminate residual confounding due to age, sex, smoking and centre using logistic regression because matching was not completely achieved on recruitment. Further, to assess the sensitivity of the analysis, we examined the adjusted odds ratios, for individual SNP allele distributions relative to the common allele, for each centre separately adjusting for age, sex and smoking. Pairwise linkage disequilibrium coefficients were calculated in controls using Haploview [9].

Because there are significant differences in allele and genotype frequencies between cases and controls for the IREB2 gene, using FAMHAP18 [10,11] we examined all possible two SNP and three SNP haplotypes for SNPs 1, 2 and 4 to explore if the haplotypes present significantly stronger associations than single SNPs. We tested the association of major and minor alleles with disease severity, comparing each of the GOLD Stages II, III and IV with Controls for TGF1 gene (SNPs 2, 3 and 4) and for IREB2 gene (SNPs 1, 2 and 4). The chi-square test of association of Major and Minor Alleles with severity was done using SAS PROC FREQ software. An apparent trend in the IREB2 gene for SNP allele frequency with increasing severity was tested using SAS PROG GLM software with CONTRAST statement. Using PROC GLM in SAS, the quantitative trait associations between SNPs and the phenotype FEV1 were tested by multivariate regression, adjusting for age, sex, smoking and centre.

## Results

The study population characteristics for those subjects with successful genotyping are summarised in Table 1. Despite attempts to match cases and controls there were significant differences observed and adjustments were made by logistic regression to take this into account in the statistical analysis. The characteristics of recruited COPD patients across the GOLD severity categories are shown in Table 2. Out of 989 duplicate genotyping reactions there were 4 discordant results (0.4%). The samples genotyped by alternative methods were 100% concordant, satisfying criteria for the assays to be accepted for further analysis.

**Table 1:** Characteristics of Controls and COPD subjects

	Control	COPD	P-value
**Male (%)**	63.9	70.0	0.0049
**Age (yr)**	60.8 ± 8.9	65.9 ± 8.2	<0.0001
**Smoking Pack Years**	38.6 ± 17.3	48.8 ± 22.9	<0.0001
**Predicted FEV**_**1 **_**(%)**	95.3 ± 10.9	43.1 ± 15.1	<0.0001
**FEV**_**1**_**/FVC (%)**	77.8 ± 4.9	47.5 ± 12.1	<0.0001
**N**	900	1002	

Values indicated are means ± SD. Pack Years missing for 6 COPD Cases.

**Table 2:** Characteristics of COPD patients according to GOLD classification of disease severity

GOLD Status	Moderate II	Severe III	Very Severe IV	P-value
**Male (%)**	74.9	67.1	67.6	0.0461
**Age (yr)**	66.0 ± 7.9	66.6 ± 8.4	64.3 ± 8.0	0.0021
**Pack Years**	49.5 ± 21.3	48.4 ± 22.4	48.6 ± 26.0	0.8043
**Predicted FEV**_**1 **_**(%)**	60.3 ± 5.9	39.9 ± 5.8	23.5 ± 4.3	<0.0001
**FEV**_**1**_**/FVC (%)**	57.1 ± 7.8	45.9 ± 9.9	36.2 ± 9.4	<0.0001
**N**	350	414	238	

Values indicated are means ± SD. Pack years missing for 3, 2, 1 persons per classification respectively.

**Figure 1:**
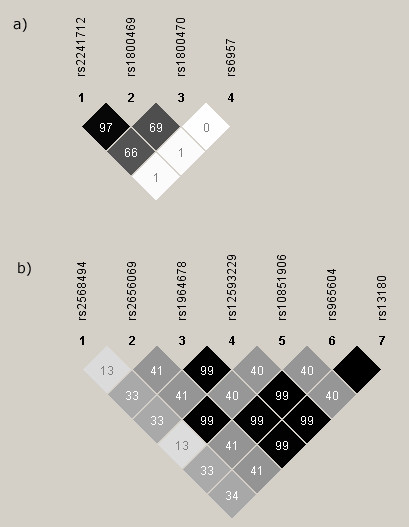
**Pairwise linkage disequilbrium between SNPs in the *TGFB1 *gene (a) and *IREB2 *gene (b)**. Values shown are for r^2^.

The values for linkage disequilibrium (LD) between the SNPs are shown in Figure 1. This reveals very strong levels of linkage disequilibrium between groups of SNPs in both genes. Within *TGFB1 *there is strong LD between SNPs 1 and 2 (rs2241712 and rs1800469; r^2 ^= 0.97), which is reflected in the very similar odds ratios calculated as part of the published meta-analysis. Within *IREB2*, it is clear that the seven SNPs studied fall into three main groups. SNPs 2 and 5 are in linkage disequilibrium (rs2656069 and rs10851906; r^2 ^= 0.99), as are SNPs 3, 4, 6 and 7 (rs1964678, rs12593229, rs965604 and rs13180; all pairwise r^2 ^= 0.99). SNP 1 (rs2568494) is not in linkage disequilibrium with any of the other *IREB2 *SNPs (r^2 ^<0.34). For this reason, subsequent tables show information for *TGFB1 *SNPs 2, 3 and 4, and *IREB2 *SNPs 1, 2 and 4 only. There are 51 SNPs in the region of *IREB2 *in HapMap, and using the 3 tagSNPs as presented here captures 64% of the variation (33/51 variants). These calculations are harder to do for *TGFB1 *as two of the SNPs we have used are not in HapMap, so we cannot calculate the LD between these SNPs and other variants in HapMap. There are only 8 SNPs in the *TGFB1 *region in HapMap, and rs1800469 captures information about one additional variant, ie 2 out of 8 SNPs (25%). It is likely that we have captured more variation than this, but are unable to quantify it more precisely due to the lack of information about our other two tagSNPs in HapMap.

HWE analysis showed that all SNPs are in HWE (p > 0.01 for all SNPs) (Table 3). The allele frequencies for SNPs in cases and controls are shown in Table 4. None of the *TGFB1 *SNPs showed significant associations with COPD. This is in contrast to the significant associations observed in the recent meta-analysis, which indicated that the minor alleles of rs2241712, rs1800470 and rs1800469 were associated with a reduced risk for COPD, whilst rs6957 was associated with increased risk [2]. Haplotype analysis also failed to demonstrate any significant association with COPD (Additional file 1: Table S1).

**Table 3:** Characteristics of the SNPs

SNP			Location in Gene	Call rate (%)	HWE p value in controls
TGFB1	1	rs2241712	5' region	94.4	0.470
	2	rs1800469	5' region	96.9	0.731
	3	rs1800470	Exon 1 (Pro10Leu)	96.5	0.782
	4	rs6957	3' region	95.5	0.593
					
IREB2	1	rs2568494	Intronic	96.6	0.450
	2	rs2656069	Intronic	96.7	0.470
	3	rs1964678	Intronic	96.2	0.778
	4	rs12593229	Intronic	97.0	0.802
	5	rs10851906	Intronic	94.1	0.460
	6	rs965604	Intronic	96.0	0.923
	7	rs13180	Exon 21 (Ala872Ala)	94.9	0.935

**Table 4:** *TGFΒ1 *and *IREB2 *Allele frequencies and odds ratios in Controls and Cases

Gene	SNP **No**.	Locus	Major/ Minor Alleles	Minor Allele Frequencies		*Unadjusted * P-value	Adjusted P-value*	Adjusted Odds Ratio** (95% C. I.)
				**Controls**	**COPD** **(all** **cases)**			
***TGFB1***	**2**	rs1800469	C/T	0.32	0.32	0.8852	0.8314	1.02 (0.87, 1.18)
	**3**	rs1800470	T/C	0.40	0.40	0.9118	0.8901	0.99 (0.86, 1.15)
	**4**	rs6957	A/G	0.18	0.17	0.2736	0.2590	0.90 (0.74, 1.08)
								
***IREB2***	**1**	rs2568494	G/A	0.35	0.41	<0.0001	0.0005	1.30 (1.12, 1.50)
	**2**	rs2656069	A/G	0.21	0.16	0.0004	0.0045	0.77 (0.64, 0.92)
	**4**	rs12593229	G/T	0.39	0.32	<0.0001	0.0013	0.79 (0.68, 0.91)

* p-values adjusted by logistic regression for age, sex, smoking and centre.

** Adjusted odds ratios are relative to the major Allele, adjusted by logistic regression for age, sex, smoking and centre.

Each of the three IREB2 SNPs showed a significant difference in allele frequency between cases and controls (Table 4). A Sidak correction for multiple testing of 6 SNPs shows that a single p-value ≤ 0.0085 corresponds to an overall p-value ≤ 0.05 [12]. Thus the significant effects remained after adjustment for multiple comparisons. The genotype frequencies and associated odds ratios for the *IREB2 *SNPs are shown in Table 5. Again the genotype differences between cases and controls are significant after the Sidak correction. This is in agreement with previously published work [6]. Haplotype analysis using IREB2 SNPs 1, 2 and 4 failed to yield any significantly stronger associations than the single SNPs alone (Additional file 1: Table S2).

**Table 5:** *IREB2 *Genotype frequencies and odds ratios in Controls and Cases.

SNP No	Locus	Genotypes	Controls	COPD all Cases	P-value		**Odds Ratio**^**+**^		
					Unadjusted	Adjusted*	Unadjusted	Adjusted ** (95% C.I.)	
1	rs2568494	GG	42.2	35.0	0.0004	0.0018	1	1	
		GA	46.4	48.4			1.26	1.24	(1.00, 1.55)
		AA	11.4	16.6			1.77	1.77	(1.29, 2.44)
2	rs2656069	AA	63.1	69.3	0.0006	0.0039	1	1	
		AG	32.2	28.7			0.81	0.85	(0.68, 1.06)
		GG	4.8	2.0			0.37	0.37	(0.20, 0.69)
4	rs12593229	GG	36.9	45.6	0.0001	0.0053	1	1	
		GT	48.0	43.9			0.74	0.79	(0.63, 0.98)
		TT	15.1	10.5			0.56	0.61	(0.44, 0.84)

* p-values adjusted by logistic regression for age, sex, smoking and centre.

^+ ^Odds ratios are relative to the major homozygous Genotype.

** Adjusted by logistic regression for age, sex, smoking and centre.

We also analysed allele frequency differences between each of the GOLD Stages II, III and IV and controls. No significant differences or trends were observed for any of the TGF1 SNPs. *IREB2 *SNPs 2 and 4 showed differences between severity groups and controls (not significant after correction for multiple testing), but there were no trends with increasing severity and the results just reflect the overall case-control relationship (Table 6). Whilst there appears to be a trend for an increase in SNP 1 minor allele frequency with increasing severity, this fails to reach statistical significance. Confining the analysis to cases only we examined the relationship between the allele distributions of the six SNPs and severity of disease using the quantitative trait FEV1 as a severity measure. We found no significant relationships between FEV1 and the alleles.

**Table 6:** Minor Allele frequencies by case, control and severity status for IREB2 SNPs 1, 2 and 4

SNP **No**.		All Controls	All Cases	GOLD II	GOLD III	GOLD IV	p-value^*^
**1**	rs2568494	34.6	40.8	38.6	41.2	43.3	0.2751
**2**	rs2656069	20.8	16.3	16.6	18.2	12.6	0.0367
**4**	rs12593229	39.1	32.4	30.7	35.2	30.0	0.0828

p-value* is the chi-square test of association of Major and Minor Alleles with severity.

Though there were significant differences between centres for the allele distribution of some of the SNPs, our results are unlikely to be due to population stratification since cases and controls were matched within centres. We also examined the genotype odds ratios for all six SNPs in each centre (Additional file 1: Table S3). The within-centre odds ratios for the three *TGFB1 *SNPs were all non-significant, showed no particular pattern and fluctuated around the overall (all-centre) odds ratios. The within-centre analysis for the *IREB2 *SNPs showed that, though non-significant due to the smaller sample size, the direction of the relationship was the same in each centre as the all-centre results. Thus each centre is showing the same IREB2 SNP relationships with COPD.

## Discussion

This study has replicated the associations seen for polymorphisms in *IREB2 *which have been previously reported, but failed to reproduce the associations seen in a recent meta-analysis for *TGFB1*. Recent studies using cluster analysis suggest that SNP rs1800470 in *TGFB1 *is associated with the emphysema-predominant phenotype [13]. There is a plausible biological explanation for this based on the critical role that *TGFB1 *plays in reducing matrix metalloproteinase-12 activity, a key mediator of emphysematous change. This is further supported by recent replicated studies showing that MMP-12 variants are associated with a protective effect on lung function and COPD [14,15]. The supposition is that low levels of MMP-12 reduce the likelihood of emphysematous change. Over-expression of *TGFB1 *would therefore predictably have the same protective effect and reduced expression would be a predisposing factor. This is also supported by work in a murine model of COPD, where activation of the TGF-Β signalling pathway was observed alongside a partial rescue of the emphysema phenotype [16]. We did not have phenotypic data to assess emphysema scores and so were unable to test this proposition. It is also possible that other genetic variants in the *TGFB1 *region which were not investigated as part of the current study may be involved in COPD susceptibility.

We have confirmed previous observations which suggest that *IREB2 *variants may play a role in COPD. This appears to be independent of an effect on lung function, as we failed to detect any association between FEV1 and any of the SNPs included in this study. This is in agreement with the genome-wide association study which looked for variants associated with lung function, which also fails to find evidence for a role of *IREB2 *[17]. The *IREB2 *gene is located within the region identified by the COPD GWAS, and the high levels of linkage disequilibrium in this area make it difficult to identify the specific functional variant or gene which is underlying this association. Only SNP 1 (rs2568494) is in relatively high levels of linkage disequilibrium with SNPs identified by the genome-wide study of COPD (HapMap CEU data: r^2 ^= 0.790 with rs8034191 and r^2 ^= 0.692 with rs1051730), although the other IREB2 SNPs are also in LD with variants in other genes in the region, including *CHRNA5*. Identification of the genes and variants contributing to the association with COPD will require targeted resequencing of the region and further functional work. In support of *IREB2 *involvement, a previous publication has shown increased levels of *IREB2 *mRNA in COPD patients versus controls [6]. This is an intriguing observation as this gene codes for an iron binding protein. Iron homeostasis and free iron concentration are likely to be important mediators of oxidative stress and iron could therefore contribute to local damage by this mechanism. IREB2 protein is expressed in the lungs and cigarette smoking has been associated with higher levels of iron in the lung [18]. *IREB2 *knock-out mouse models have a predisposition to developing neurodegenerative disease due to aberrant cellular iron homeostasis [19], though the lungs of these animals were not examined in any detail. A potential mechanism for distorted ion homeostasis relates to membrane serine proteases (matriptases) which regulate a number of biological effects. Genome-wide association studies have shown that mutations in matriptase result in refractory iron deficiency anaemia and the proteolytic activity of matriptase is critical in regulating iron in this form of anaemia as loss of function mutations result in the disease [20]. The catalytic domain of matriptase is inhibited by alpha1-antitrypsin [21] and it is therefore conceivable that two major pathways associated with progressive lung damage, namely proteolysis and oxidative stress, are linked through iron homeostasis. This could also explain the tendency for greater oxidative stress in alpha1-antitrypsin deficiency as more active matriptase may result in excessive free iron.

## Conclusions

In summary, this study failed to replicate previous reports of associations between SNPs in *TGFB1 *and susceptibility to COPD, despite being adequately powered. This may be due to the possibility that the association is actually with specific emphysema phenotypes rather than overall susceptibility to disease, which could not be assessed in this patient group. The replication of the association between variants of *IREB2 *and COPD provides further evidence to support the role of this genomic region in COPD pathogenesis, and the role of iron regulation deserves further investigation.

## Competing interests

The authors declare that they have no competing interests.

## Authors' contributions

SC, JL, LD, COC and NK made substantial contribution to the conception and design of the study, and analysis and interpretation of the data. AA carried out the duplicate genotyping as part of the quality control measures.

KM, TGB, JR, RR, ABM, SCD, VK, WM, JS, PSH, MM and SM made a substantial contribution to the collection of the resource and an intellectual contribution to the study design. All authors read and approved the final manuscript.

## Pre-publication history

The pre-publication history for this paper can be accessed here:

http://www.biomedcentral.com/1471-2350/12/24/prepub

## Supplementary Material

Additional file 1 **Supplementary Tables**. Table S1: TGFB1 haplotype frequencies in Cases and Controls. Table S2: IREB2 haplotype frequencies in Cases and Controls. Table S3: TGF1 and IREB2 Allele frequencies and odds ratios in Cases and Controls by Centre Click here for file
