# Supplementary material for: The role of IREB2 and transforming growth factor beta-1 genetic variants in COPD: a replication case-control study
Source: BMC Med Genet. 2011 Feb 14;12:24. doi: 10.1186/1471-2350-12-24 (PMC3047296; doi:10.1186/1471-2350-12-24)
Supplement: Additional file 1 — Supplementary Tables. Table S1: TGFB1 haplotype frequencies in Cases and Controls. Table S2: IREB2 haplotype frequencies in Cases and Controls. Table S3: TGF1 and IREB2 Allele frequencies and odds ratios in Cases and Controls by Centre [file 1471-2350-12-24-S1.DOCX]

**Supplementary Table 1: TGFB1 haplotype frequencies in Cases and Controls**

|  |  |  | Frequency (%) | |  |  |
| --- | --- | --- | --- | --- | --- | --- |
| Gene | SNP | Haplotypes | COPD  all Cases | Controls | Odds Ratios^*^ | p-value^+^ |
| TGFB1 | 2 & 3 | C-T | 0.60 | 0.60 | 0.98 | 0.7205 |
|  |  | C-C | 0.08 | 0.08 | 1.01 |  |
|  |  | T-C | 0.31 | 0.31 | 1.01 |  |
|  |  | T-T | 0.00 | 0.00 |  |  |
|  |  |  |  |  |  |  |
|  | 2 & 4 | C-A | 0.59 | 0.58 | 1.04 | 0.5364 |
|  |  | C-G | 0.09 | 0.10 | 0.89 |  |
|  |  | T-A | 0.25 | 0.24 | 1.05 |  |
|  |  | T-G | 0.07 | 0.08 | 0.90 |  |
|  |  |  |  |  |  |  |
|  | 3 & 4 | T-A | 0.51 | 0.50 | 1.04 | 0.6597 |
|  |  | C-A | 0.32 | 0.32 | 1.03 |  |
|  |  | T-G | 0.09 | 0.10 | 0.90 |  |
|  |  | C-G | 0.08 | 0.08 | 0.93 |  |
|  |  |  |  |  |  |  |
|  | 2 & 3 & 4 | C-T-A | 0.51 | 0.50 | 1.04 | 0.8345 |
|  |  | C-C-A | 0.08 | 0.08 | 1.00 |  |
|  |  | C-T-G | 0.09 | 0.10 | 0.88 |  |
|  |  | T-C-A | 0.24 | 0.24 | 1.04 |  |
|  |  | T-T-A | 0.00 | 0.00 |  |  |
|  |  | T-C-G | 0.07 | 0.08 | 0.91 |  |
|  |  | C-C-G | 0.01 | 0.01 | 1.03 |  |

^*^ Odds ratios are relative to the other haplotypes.

^+^ p-values are by Monte Carlo simulation on 10,000 runs

**Supplementary Table 2: IREB2 haplotype frequencies in Cases and Controls**

| Gene | SNP | Haplotypes | Frequency (%) | | Odds Ratios* | p-value^+^ |
| --- | --- | --- | --- | --- | --- | --- |
|  |  |  | COPD  all Cases | Controls |  |  |
| IREB2 | 1 & 2 | G-A | 0.43 | 0.44 | 0.94 | 0.0004 |
|  |  | A-A | 0.41 | 0.35 | 1.30 |  |
|  |  | G-G | 0.17 | 0.21 | 0.74 |  |
|  |  | A-G | 0.00 | 0.00 |  |  |
|  |  |  |  |  |  |  |
|  | 1 & 4 | G-G | 0.26 | 0.26 | 1.00 | < 0.0001 |
|  |  | A-G | 0.41 | 0.35 | 1.32 |  |
|  |  | G-T | 0.33 | 0.39 | 0.75 |  |
|  |  | A-T | 0.00 | 0.00 |  |  |
|  |  |  |  |  |  |  |
|  | 2 & 4 | A-G | 0.68 | 0.61 | 1.33 | 0.0005 |
|  |  | A-T | 0.16 | 0.18 | 0.87 |  |
|  |  | G-T | 0.16 | 0.21 | 0.73 |  |
|  |  | G-G | 0.00 | 0.00 |  |  |
|  |  |  |  |  |  |  |
|  | 1 & 2 & 4 | G-A-G | 0.27 | 0.26 | 1.01 | 0.0003 |
|  |  | A-A-G | 0.41 | 0.35 | 1.31 |  |
|  |  | G-A-T | 0.16 | 0.18 | 0.87 |  |
|  |  | G-G-T | 0.16 | 0.21 | 0.74 |  |
|  |  | A-A-T | 0.00 | 0.00 |  |  |
|  |  | A-G-G | 0.00 | 0.00 |  |  |
|  |  | G-G-G | 0.00 | 0.00 |  |  |

^*^ Odds ratios are relative to the other haplotypes.

^+^ p-values are by Monte Carlo simulation on 10,000 runs

## Supplementary Table 3: TGF1 and IREB2 Allele frequencies and odds ratios in Cases and Controls by Centre

| **Centre ^+^** | **Gene** | **SNP**  **No.** | **Locus** | **Major/**  **Minor**  **Alleles** | **Minor Allele**  **frequencies** | | **Adjusted**  **Odds Ratio***  **(95% C.I)** |
| --- | --- | --- | --- | --- | --- | --- | --- |
|  |  |  |  |  | **All Cases** | **Controls** |  |
| 1 | ***TGFB1*** | 2 | rs1800469 | C/T | 0.33 | 0.30 | 1.01 (0.61, 1.68) |
| 2 |  |  |  |  | 0.25 | 0.25 | 1.08 (0.67, 1.75) |
| 3 |  |  |  |  | 0.29 | 0.26 | 1.34 (0.95, 1.90) |
| 4 |  |  |  |  | 0.32 | 0.30 | 1.13 (0.71, 1.77) |
| 5 |  |  |  |  | 0.29 | 0.33 | 0.82 (0.60, 1.13) |
| 6 |  |  |  |  | 0.41 | 0.43 | 0.94 (0.69, 1.26) |
| 1 |  | 3 | rs1800470 | T/C | 0.40 | 0.37 | 0.99 (0.61, 1.62) |
| 2 |  |  |  |  | 0.34 | 0.32 | 1.09 (0.70, 1.69) |
| 3 |  |  |  |  | 0.39 | 0.34 | 1.32 (0.96, 1.82) |
| 4 |  |  |  |  | 0.41 | 0.41 | 1.22 (0.70, 1.88) |
| 5 |  |  |  |  | 0.35 | 0.41 | 0.78 (0.58, 1.05) |
| 6 |  |  |  |  | 0.47 | 0.51 | 0.86 (0.63, 1.15) |
| 1 |  | 4 | rs6957 | A/G | 0.19 | 0.19 | 0.83 (0.45, 1.53) |
| 2 |  |  |  |  | 0.14 | 0.19 | 0.75 (0.42, 1.31) |
| 3 |  |  |  |  | 0.18 | 0.19 | 1.05 (0.70, 1.57) |
| 4 |  |  |  |  | 0.16 | 0.19 | 0.79 (0.45, 1.38) |
| 5 |  |  |  |  | 0.13 | 0.18 | 0.71 (0.48, 1.06) |
| 6 |  |  |  |  | 0.18 | 0.17 | 1.17 (0.79, 1.73) |
| 1 | *IREB2* | 1 | rs2568494 | G/A | 0.40 | 0.35 | 1.04 (0.64, 1.69) |
| 2 |  |  |  |  | 0.41 | 0.35 | 1.51 (0.97, 2.35) |
| 3 |  |  |  |  | 0.34 | 0.32 | 1.02 (0.73, 1.41) |
| 4 |  |  |  |  | 0.44 | 0.31 | 1.99 (1.28, 3.10) |
| 5 |  |  |  |  | 0.38 | 0.32 | 1.32 (0.98, 1.79) |
| 6 |  |  |  |  | 0.48 | 0.41 | 1.29 (0.96, 1.74) |
| 1 |  | 2 | rs2656069 | A/G | 0.13 | 0.21 | 0.81 (0.43, 1.50) |
| 2 |  |  |  |  | 0.13 | 0.19 | 0.50(0.27, 0.91) |
| 3 |  |  |  |  | 0.19 | 0.21 | 0.94 (0.64, 1.39) |
| 4 |  |  |  |  | 0.16 | 0.17 | 0.79 (0.45, 1.37) |
| 5 |  |  |  |  | 0.18 | 0.22 | 0.75 (0.52, 1.08) |
| 6 |  |  |  |  | 0.17 | 0.22 | 0.75 (0.51, 1.08) |
| 1 |  | 4 | rs12593229 | G/T | 0.32 | 0.39 | 1.00 (0.60, 1.66) |
| 2 |  |  |  |  | 0.30 | 0.35 | 0.74 (0.47, 1.17) |
| 3 |  |  |  |  | 0.35 | 0.40 | 0.93 (0.68, 1.27) |
| 4 |  |  |  |  | 0.32 | 0.39 | 0.75 (0.48, 1.16) |
| 5 |  |  |  |  | 0.34 | 0.40 | 0.76 (0.56, 1.02) |
| 6 |  |  |  |  | 0.30 | 0.41 | 0.67 (0.49, 0.91) |

^+^ Centres are: 1= Barcelona, 2 = Bristol, 3 = Dublin, 4 = Edinburgh, 5 = Leiden and 6 = Pisa.

* Adjusted odds ratios are relative to the major allele, adjusted by logistic regression for age, sex and smoking.
